# Perinatal Depression and Associated Factors among Mothers in Southern Ethiopia: Evidence from Arba Minch Zuria Health and Demographic Surveillance Site

**DOI:** 10.1155/2018/7930684

**Published:** 2018-05-02

**Authors:** Gebrekiros Gebremichael, Manaye Yihune, Dessalegn Ajema, Desta Haftu, Genet Gedamu

**Affiliations:** College of Medicine and Health Sciences, Arba Minch University, Arba Minch, Ethiopia

## Abstract

*Background. *Perinatal depression is a serious mental health problem that can negatively affect the lives of women and children. The adverse consequences of perinatal depression in high-income countries also occur in low-income countries.* Objective.* To assess the perinatal depression and associated factors among mothers in Southern Ethiopia.* Methods.* A community based cross-sectional study was conducted among selected 728 study participants in Arba Minch Zuria HDSS. A pretested questionnaire was used to collect the data. Data were analyzed using STATA version 12 software. Descriptive statistical methods were used to summarize the characteristics of the mothers. Bivariate and multivariable logistic regression was used for analysis.* Results.* The prevalence of perinatal depression among the study period was 26.7%. In the final multivariable logistic regression, monthly income AOR (95% C.I): 4.2 (1.9, 9.3), parity [AOR (95% C.I): 0.14 (0.03, 0.65)], pregnancy complications AOR (95% C.I): 5 (2.5, 10.4), husband smoking status [AOR (95% C.I): 4.12 (1.6, 10.6)], history of previous depression AOR (95% C.I): 2.7 (1.54, 4.8), and family history of psychiatric disorders were the independent factors associated with perinatal depression.* Conclusion.* The study showed a high prevalence of perinatal depression among pregnant mothers and mothers who have less than a one-year-old child.

## 1. Introduction

Depression is a common, treatable mood disorder. About 6% of women, including up to 10% of women who are pregnant, will experience depression at some time during their lives. Women are more at risk of depression while they are pregnant, and during the weeks and months after having a baby [1, 2].

Perinatal depression refers to major and minor depressive episodes that occur either during pregnancy (antenatal depression) or in the first 12 months after delivery (postnatal depression) [3, 4]. Antenatal depression is a major health problem but is less well-studied than postpartum depression [5].

Perinatal depression affects more than 20% of pregnant women and mothers. As many as 14.5% of new mothers in the United States suffer from major depression. Unipolar depression makes a large contribution to the burden of disease, being at third place worldwide and eighth place in low-income countries, but at first place in middle- and high-income countries. Depression is the leading cause of disease burden for women in both high-income and low- and middle-income countries [6, 7].

The incidence of perinatal depression varies with the population surveyed, but estimated rates for depression among pregnant and postpartum women have ranged from 5% to 25%. Studies of low-income mothers and pregnant and parenting teenagers have reported rates of depressive symptoms at 40% to 60% [8] and mean prevalence of 15.6% in pregnant women and 19.8% in women who had given birth recently [9]. In general, as many as 12% of all pregnant or postpartum women experience depression in a given year, and for low-income women the prevalence is doubled [8].

The prevalence of perinatal depression was 31.5% among mothers in a community based cross-sectional study from southeast Ethiopia [10]. Another population based cohort study in Ethiopia reported that the prevalence of perinatal depression was 13.8%, of which 9.2%, 2.1%, and 2.5% of the women were depressed antenatally, postnatally, and persistently, respectively [11].

Studies have identified a number of practical, psychological, and cultural barriers to mental health service use by low-income populations including cost, inconvenient clinic locations, transportation, limited hours, childcare, stigma, discrimination, previous negative treatment experiences, burden of depression, and the provider's cultural insensitivity [12–15].

Perinatal depression is a serious mental health problem that can negatively affect the lives of women, children, and their families. Research in developing countries suggests that poor maternal mental health, in particular perinatal depression, may be a risk factor for poor growth in young children. In low-income countries, the prevalence of perinatal depression is estimated at 15–25% [15–18].

The risk of depression in women is approximately twofold higher than in men and women are particularly prone in the postpartum period because of hormonal changes associated with childbirth and stressors associated with parenting. The combination of women's vulnerability to depression, their responsibility for childcare, and the high prevalence of perinatal depression in developing countries could have a substantial influence on growth during childhood [13, 19, 20].

The adverse consequences found for children of depressed mothers in high-income countries also occur in low- and middle-income countries, including difficult temperament, behavior problems, and deficits in cognitive performance and academic achievement. Health consequences include poor physical growth and an increased risk of gastrointestinal illness. In addition, in low- and middle-income countries, perinatal depression also increases the risk for paternal depression. Common mental disorders symptoms are also independently associated with functional impairment in both pregnancy and the postnatal period [14, 21].

In Ethiopia, although there are some studies especially on postpartum depression, depression during the perinatal period is not studied well including in the study area. Thus, this study aims to examine the prevalence and the associated factors of perinatal depression in Arba Minch Zuria Health and Demographic Surveillance Site, Southern Ethiopia.

## 2. Methods

### 2.1. Study Area and Period

The study was conducted in Arba Minch Zuria District, Gamo Gofa Zone, Southern Ethiopia. Arba Minch Zuria District has 31 kebeles where 29 are rural and 2 are semiurban. Besides there are three climatic zones classified as lowland, midland, and highland. The total population of the District was 165,680 of which 82,751 were males and 82,929 were female according to the central statistical agency 2007 report. Arba Minch Zuria Health and Demographic Surveillance Site (HDSS) was established in 2009 in collaboration with Center for Disease Control (CDC), Ethiopian Public Health Association (EPHA), and Arba Minch University. Stratification was made based on the climatic zone and urbanization of the kebeles; then random cluster sampling was employed. Purposive sampling technique was used to select the urban kebele. Accordingly, nine kebeles were selected where 8 of the kebeles are rural and the remaining one kebele was semiurban; based on climatic zone two of them are highlands, four of them are lowlands, and the remaining three are midlands. The study was conducted from April 15 to May 30, 2017.

### 2.2. Study Design

A community based cross-sectional study design was implemented.

### 2.3. Source Population and Study Population

All pregnant mothers and mothers having a child less than one year in Arba Minch Zuria District were the source population and selected pregnant mothers and mothers having a child less than one year in Arba Minch Zuria HDSS kebeles were the study population.

### 2.4. Sample Size Determination and Sampling Procedures

Sample size was calculated for both specific objectives using the Epi Info version 7 software and considering different parameters (95% confidence level, proportion of perinatal depression = 31.5% [10], 5% margin error, 2 deign effect, and 10% nonresponse rate), and then a final sample size of 728 was used.

The nine kebeles included in the Arba Minch Zuria HDSS were selected by stratification based on the climatic zone and urbanization of the kebeles, and then random cluster sampling was employed. Accordingly, nine kebeles were selected where 8 of the kebeles are rural and the remaining one kebele was semiurban; based on climatic zone two of them are highlands, four of them are lowlands and the remaining three are midlands.

Study participants were then allocated nonproportionally to the number of pregnant mothers and mothers having a child less than one year (nonproportional to size) in each kebele. Finally, sampling frame was prepared for each kebele and simple random sampling technique was applied to select the study participants.

### 2.5. Study Variables

#### 2.5.1. Dependent Variable

Perinatal depression is the dependent variable.

#### 2.5.2. Independent Variables

*Sociodemographic Characteristics. *Age, educational status, economic status, marital status, and employment are the sociodemographic characteristics.

*Psychosocial Support*. Husband support, domestic violence, childbirth without the presence of any relatives, relationship in marriage, and perceived difficult of childcare are the psychosocial support variables.

*Substance Use*. Maternal and husband substance use of alcohol, khat, and cigarettes is one of the independent variables.

*Obstetric Factors*. Age at marriage, numbers of total children, plan to have child, experiences of the deaths of child parity, unplanned pregnancy, losing or hospitalizing a baby, mode of delivery, pregnancy complication or illness, stressful life event during pregnancy, and fetal sex are the obstetric factors.

*Previous Psychiatric History*. This includes history of depression and family history of psychiatric problems.

### 2.6. Operational Definitions

#### 2.6.1. Perinatal Depression

Any symptoms of depression during the perinatal period with score below six using the SRQ-20 questionnaire refer to perinatal depression [10, 14].

#### 2.6.2. Khat

It is stimulant leaves of a shrub that have a stimulating and euphoric effect when chewed.

### 2.7. Data Collection Tools

The WHO Self-Reported Questionnaire (SRQ-20) with 20 items was used to assess the perinatal depression. The tool consists of twenty Yes/No questions with a reference period of the previous thirty days. It has acceptable levels of reliability and validity in developing countries and is recommended by the World Health Organization as a screening tool for depression [22].

A semistructured, interviewer administered questionnaire in Amharic language was used to collect data. The tool also includes sociodemographic variables, obstetric history, maternal history of pervious mental illness, family history of mental diseases, and the presence of social support during the perinatal period and husband and maternal substance use.

The SRQ-20 questionnaire is validated in Ethiopia for use in a mixed sample of pregnant and postnatal women. As a result, the overall performance of the SRQ-20 did not differ significantly between pregnant and postnatal women and have superior validity to the EPDS across all domains for evaluating cultural equivalence in detection of perinatal common mental disorders [14]. This tool had good internal consistency (Cronbach's alpha = 0.83) in this study.

Pretest was conducted two weeks prior to the actual data collection on Mirab Abaya District, and modification was made accordingly. Nine trained female data collectors were recruited.

### 2.8. Data Quality Assurance

The quality of the data was maintained before, during, and after the data collection. Before the data collection designing/adapting of structured standard questionnaire, four-day training of data collectors and supervisors about the objective, questionnaire, methodology, and ethical issues of the study and pretesting of the questionnaire were undertaken.

During the data collection period, the collected data were checked for completeness and consistencies by the supervisors and the investigator through close follow-up. Missed variable/s during the first visit were filled by reinterviewing the study participants.

After the data collection, the collected data were rechecked for their completeness and consistency by the investigators. Then, they were entered into Epi Info version 7; then 5% of the data set were double-entered to check the accuracy of the entered data.

### 2.9. Data Management and Analysis

Data were coded, stored in a proper area and kept confidential, and then entered into a prepared Epi info template. The data were exported to STATA version 12 for cleaning and analysis. The data were cleaned by running simple frequency and cross tabulation to check for completeness and consistency and sorting to identify outliers.

Data were analyzed using STATA version 12; odds ratio with 95% confidence interval was used to measure the association between perinatal depression and the independent (sociodemographic, obstetric, substance abuse, and psychosocial support) variables.

For specific objective one, descriptive statistical methods such as frequencies, percentages, proportion with 95% CI, and mean and standard deviation were used to summarize the various characteristics of the mothers.

For specific objective two, cross tabulation and bivariate logistic regression were used to explore the relation between perinatal depression and the different independent variables using crude odds ratio with 95% CI. Finally, to determine the independent factors associated with perinatal depression, multivariable logistic regression model was done and presented using Adjusted ORs (AOR) with 95% Confidence Interval.

Variables with *P* value < 0.05 in the bivariate analysis were taken to the multivariable logistic regression model. Model fitting was checked using log likelihood and Hosmer-Lemeshow test (i.e., Hosmer-Lemeshow test of the model was nonsignificant with *P* value 0.24). Finally, variables with *P* < 0.05 in the multivariable analysis were considered as significant.

## 3. Result

A total of 704 study participants were involved in this study, yielding a 96.7% response rate. The rest of the respondents did not participate due to refusal and absence during the data collection period, and some questionnaires were discarded due to incompleteness.

### 3.1. Sociodemographic Characteristics

Majority of the participants were in age groups between 25 and 34 years (54.55%), protestant in religion (72.44%), from Gamo ethnicity (66%), married (96.58%), with unable to read and write educational status (51.14%), and housewives (76.7%). The mean ± SD age of the respondents was 28.02 ± 5.8 with 16 and 42 the minimum and maximum years, respectively. Regarding the husband's characteristics, majority of them were farmers (69%) and had lower educational status. Forty-one percent and about 38% of the study participants had less than 1000 ETB and did not know their monthly income, respectively (Table 1).

### 3.2. Obstetric Characteristics

The study participants had an average of 3.5 number of pregnancies with minimum of 1 and maximum of 9 pregnancies. Seventeen percent (119) of the study participants were primigravida, 51.7% (362) were multigravida, and 31.3% (219) were grand multigravidas. In addition to this, the majority (57.6%) of the study participants had two to four children followed by one (19.5%), more than four (13.9%), and no (9%), children respectively.

The mean age of the mothers at marriage was 20.2±3 years, and the minimum and maximum ages were 14 and 30 years, respectively. Concerning the abortion history of the study participants, 18.6% (131) of them reported that they had history of abortion, whereas the rest 81.4% (573) had no abortion history. Among the mothers who had abortion, 86.3% (113) had abortion once and only 13.7% [18] had twice history of abortion.

Sixteen percent of the study participants reported that they had health problems related to their current or previous pregnancy. Most of them mentioned that they had malaria, peptic ulcer disease, diarrhea, anemia, hypertension, and renal infections. On the other hand, the majority (84%) of the study participants reported that they did not experience any health problems during their recent pregnancy.

**Figure 1:**
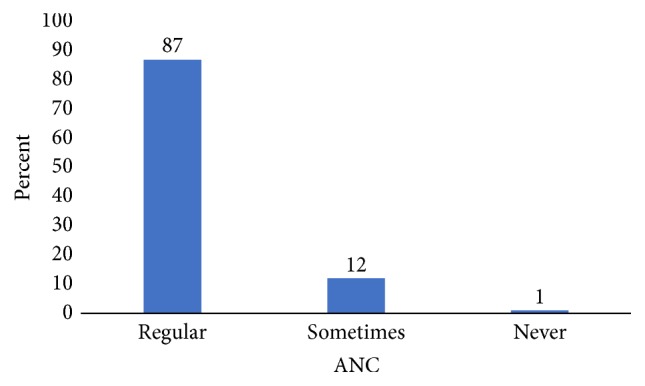
Antenatal care follow-up history of participants in Arba Minch Zuria District, Ethiopia, 2017.

About 82% (576) of the respondents reported that their pregnancy or child was planned, whereas the remaining 18% (128) of them revealed that it was not planned. The majority of the study participants (87%) had regular antenatal care (ANC) follow-up (Figure 1).

The majority of the study participants responded that they had not experienced child death (83%) and hospitalization (92%). On the contrary, 16% of them reported child death and only 8% child hospitalization. Regarding the mode of delivery of the participants recent birth, almost all (94%) delivered vaginally and the remaining six percent of the mothers through cesarean section and instrumental delivery. Fifty-three and forty-seven percent of the mothers had male and female child, respectively.

### 3.3. Substance Abuse

The respondents were asked about the alcohol, khat, and cigarette use by themselves and by their partners. Accordingly, the majority of the respondents and did not drink alcohol (71.6%), did not chew khat (98.6%), and did not smoke cigarette (98%). Similarly, 98% and 91.4% of their partners did not chew khat and did not smoke cigarettes, respectively (Table 2).

### 3.4. Previous Psychiatric Histories

Only 22.6% (159) of the study participants reported that they had previous history of depression. But the majority (77.4%) of the respondents did not have previous history of depression. Moreover, about 92% of them reported that they have no family psychiatric disorders. On the other hand, only 4.35% and 3.7% mentioned that they have near relative and distant relative, respectively, with mental disorders.

### 3.5. Psychosocial Support

**Figure 2:**
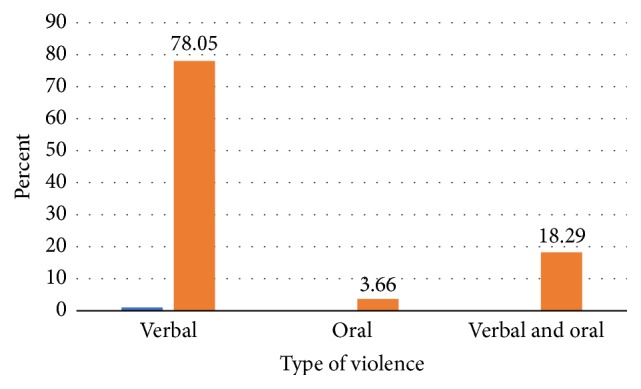
Type of violence the study participants experienced at home in Arba Minch Zuria District, Ethiopia, 2017.

Among the study participants in the study, 164 (23.3%) mothers responded that they had violence at home, whereas the remaining, 540 (76.7%), respondents did not experience violence. From the mothers who had violence at home, the majority (78%) had oral type of violence (Figure 2).

Regarding the happiness of the mothers with their marriage, the majority (86%) of them were happy, and the remaining 14% were not happy. Similarly, the respondents were asked about their partner's feeling to their recent pregnancy/child. Accordingly, 61% (426), 28.5% (200), and 10.8 (76) of their partners' feelings were very good, good, and not good, respectively.

Eighty-four percent of the study participants said that they did not perceive that they will have difficulty of caring for a child. On the other hand, 16% of them perceive that they will have difficulty of caring for a child. Regarding the husband support to the mothers and their children, the majority (85%) of the mothers reported that their husbands support them, whereas the remaining 15% did not get husband support. Almost ninety-two percent of the respondents who ever gave birth mentioned that their relatives were present in health facilities during their last childbirth. Only 8% of mothers said that none of their relatives were present. In addition to this, 90% and 10% of the study participants were happy and unhappy, respectively, with their husband families.

### 3.6. Prevalence of Depression

**Figure 3:**
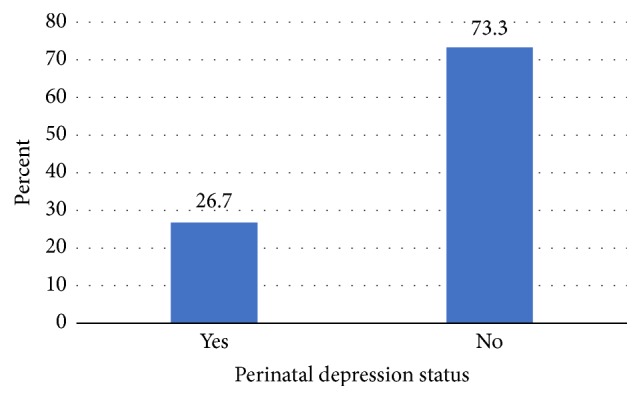
Perinatal depression status of the study participants according to the SRQ-20 in Arba Minch District, Ethiopia, 2017.

The mean score of self-reporting questionnaire is 4.22 with standard deviation of 0.14. The prevalence of depression according to the self-reporting questionnaire among the study period was 26.7% (Figure 3).

### 3.7. Factors Associated with Perinatal Depression

#### 3.7.1. Sociodemographic Factors

Among the sociodemographic characteristics of the mothers and their partners, educational status of the mothers and their partners, occupational status of the mothers and their partners, and monthly income were the significant factors with perinatal depression in the bivariate logistic regression. However, age group and marital status of the study participants showed nonsignificant association (Table 3).

Mothers who are unable to read and write had 2.86 times more odds of having perinatal depression compared to mothers having educational status of high school and above [OR (95% C.I): 2.86 (1.6, 5.1)]. Similarly, mothers whose partners could not read and write and have primary education had 1.8 and 1.7 odds of having perinatal depression, respectively, compared to those mothers whose partners are educated, secondary level and above [OR (95% C.I): 1.8 (1.08, 3), 1.7 (1.02, 2.85)].

Regarding the association between occupational status of the mothers and their partners with perinatal depression, being a house wife is associated with 1.8 more odds of perinatal depression compared to other occupations. Likewise, husband occupation of merchant is related to 2.4 more odds of perinatal depression than farmers [OR (95% C.I): 2.4 (1.4, 4)]. And other husband occupations (governmental employee, jobless) are associated with 60% lower odds of perinatal depression [OR (95% C.I): 0.4 (0.2, 0.9)].

Mothers who have lower income and who do not know their income are 2 and 4.6 times more likely to be depressed perinatally compared to mothers who have higher income [OR (95% C.I): 2 (1.15, 3.6), 4.6 (2.7, 8)].

#### 3.7.2. Obstetric Factors

In the bivariate logistic regression, number of pregnancy/children, age at marriage, abortion, health problems during pregnancy, planned pregnancy, and child death showed significant association with perinatal depression. On the contrary, factors such as antenatal care, child hospitalization, mode of delivery, and child sex were not significant (Table 4).

The bivariate logistic regression showed that the primigravida mothers had 84% times lower odds of perinatal depression compared to the grand multigravidas [OR (95% C.I): 0.16 (0.08, 0.31)]. In line with this, the multigravidas also had 57% times lower likelihood of depression than the grand multigravidas [OR (95% C.I): 0.43 (0.3, 0.6)].

Mothers who have no child, one child, and two to four children have 95%, 63%, and 32% lower probability of having perinatal depression, respectively, compared to those mothers having more than five children [OR (95% C.I): 0.05 (0.01, 0.22), 0.37 (0.2, 0.66), 0.68 (0.43, 0.97)]. Likewise, every increase of one year in age at marriage is associated with 1.1 times increase in perinatal depression OR (95% C.I): 1.1 (1.05, 1.17).

The odds of perinatal depression are 2.54 and 4.9 times higher in those respondents who had abortion and health problems during pregnancy compared to their counterparts OR (95% C.I): 2.54 (1.7, 3.8), 4.9 (3.2, 7.5). Besides, having planned pregnancy was associated with 42% lower perinatal depression [OR (95% C.I): 0.58 (0.39, 0.88)], and experiencing child death is associated with 2.3 times more perinatal depression [OR (95% C.I): 2.3 (1.5, 3.5)].

#### 3.7.3. Substance Abuse

Among the selected substance abuse related characteristics of the study participants and their partners, alcohol use of the mothers, husband khat chewing, and husband cigarette smoking showed significant association with perinatal depression in the bivariate logistic regression. On the other hand, khat, smoking history of the participants, and husband alcohol use were not significantly associated (Table 5).

Mothers who drink alcohol had 40% times lesser odds of being depressed relative to those mothers who did not drink alcohol OR (95% C.I): 0.6 (0.4, 0.9). Likewise, husband history of khat chewing and cigarette smoking was associated with 3.8 and 3.6 times higher odds of perinatal depression in the mothers, respectively [OR (95% C.I): 3.8 (1.3, 11), 3.6 (2.1, 6.2)].

#### 3.7.4. Previous History of Mental Disorders

Both previous history of depression and family history of mental disorders are significantly associated with perinatal depression of the study participants in the binary logistic regression (Table 6).

Previous history of depression in the study participants was related to 3.54 times more odds of perinatal depression compared to respondents with no history of previous depression OR (95% C.I): 3.54 (2.44, 5.15). Furthermore, study participants who had relatives with mental disorders had 3.6 and 5 times more odds of perinatal depression for near and distant relatives, respectively, as compared to those respondents who had no history of family members with mental problems [OR (95% C.I): 3.6 (1.73, 7.2), 5 (2.25, 11.4)].

#### 3.7.5. Social Support Related Factors

Experience of abuse, husband feelings about the recent pregnancy/birth, difficult perception of caring child, husband support, and presence of family at health institutions during last birth are significantly associated with perinatal depression in the binary logistic regression analysis, whereas being happy in their marriage and with their in-laws did not show significant association (Table 7).

Respondents who reported any type of abuse at home had 1.6 times more odds of perinatal depression compared to those respondents without any abuse [OR (95% C.I): 1.6 (1.2, 2.3)]. The study participants' husbands' very good and good feelings towards the recent pregnancy/birth both are negatively associated with perinatal depression of the mothers [OR (95% C.I): 0.35 (0.21, 0.58), 0.35 (0.2, 0.61)].

Perceiving difficulty of child caring is related to 3.6 times more presence of perinatal depression [OR (95% C.I): 3.6 (2.4, 5.5)]. On the other hand, husband support and family presence during the last birth at health institutions were associated with 57% and 53% times lesser odds of perinatal depression, respectively, compared to those respondents who had no husband support and no family member during birth [OR (95% C.I): 0.43 (0.28, 0.66), 0.47 (0.27, 0.83)].

#### 3.7.6. Multivariable Logistic Regression

After the bivariate logistic regression of each variable with perinatal depression, those variables which were significant were taken to the multivariable logistic regression. Besides, variables husband occupation and husband smoking behavior were dropped from the final multivariable logistic regression due to possibility of correlation with the other independent variables.

In the final multivariable logistic regression, monthly income, number of pregnancy, age at marriage, health problems during pregnancy, alcohol use, husband smoking status, history of previous depression, and family history of psychiatric disorders were the independent factors associated with perinatal depression.

However, among the sociodemographic variables, educational status of the respondents and their partners and occupational status did not show significant statistical association in the multivariable model. Besides, number of children, abortion history, planned pregnancy/birth, child death, and all of the selected social support related variables showed nonsignificant association.

Mothers who had lower income and who do not know their income had 4.2 and 5.9 times more odds of perinatal depression, respectively, compared to the mothers with relatively higher income [AOR (95% C.I): 4.2 (1.9, 9.3), 5.9 (2.6, 13.5)] (Table 3).

Likewise, mothers who are pregnant for the first time had 86% times lower odds of perinatal depression than the mothers who were pregnant for more than four times [AOR (95% C.I): 0.14 (0.03, 0.65)]. On the other hand, being pregnant for two to four times showed marginally significant association with perinatal depression after controlling all other variables (Table 4).

Every one-year increase in age at marriage is independently associated with 1.2 times increase in perinatal depression AOR (95% C.I): 1.2 (1.1, 1.3). Similarly, study participants who had health problems during their pregnancy had 5 times more likelihood of perinatal depression compared to mothers who do not experience health problems AOR (95% C.I): 5 (2.5, 10.4) (Table 4).

Study respondents who drink alcohol had 3.7 times lower odds of perinatal depression as compared to those who did not drink alcohol [AOR (95% C.I): 0.27 (0.14, 0.52)]. On the contrary, husband smoking status is independently associated with about 4 times more likelihood of perinatal depression counterpointed to husbands not smoking [AOR (95% C.I): 4.12 (1.6, 10.6)] (Table 5).

Previous history of depression and family history of mental disorders were also independently associated factors with perinatal depression. Accordingly, respondents who had previous history of depression and family history of mental disorders were associated with 2.7 and 3.6 times more chance of having perinatal depression, respectively, as compared with their counter parts [AOR (95% C.I): 2.7 (1.54, 4.8), 3.6 (1.4, 9.1)] (Table 6).

## 4. Discussion

The prevalence of perinatal depression among the pregnant mothers and mothers having less than one year in this study was high. The study reported a perinatal depression prevalence of 26.7 percent (95% C.I: 23.4, 30) using the 20 item self-reporting questionnaire (SRQ-20). This prevalence is similar to some studies conducted elsewhere [8, 18, 23–26]. However, this study showed a higher prevalence of depression than studies conducted in other low and high-income countries [4, 9, 11, 27–32], and lower compared to other studies [10, 33–38]. These disparities might be due to the differences in the depression measurement tools the various studies used, the cut-off points used, and the study settings and design.

The independent factors associated with perinatal depression in this study were monthly income, number of pregnancy, age at marriage, health problems during pregnancy, alcohol use, husband smoking status, history of previous depression, and family history of psychiatric disorders.

Mothers who had lower income and who do not know their income had 4.2 and 5.9 times more odds of perinatal depression independently compared to the mothers with relatively higher income. Previous studies had also showed consistent findings with this [29, 30, 32, 35, 37].

Likewise, mothers who are pregnant for the first time had 86% times lower odds of perinatal depression than the mothers who were pregnant for more than four times. This finding is in contrast to previous researches conducted at Northern Ethiopia and Japan [23, 39]. The possible reasons in this study that having more history of pregnancy is associated with more depression may be that having more pregnancy results in larger family size and lower income and thus making the mothers more depressed.

This study reported high rate of planned pregnancy (82%), and higher antenatal care follow-up (87%) compared to reports from different lower and middle-income countries elsewhere. This may be an indication of changes in educational status of the family members and increased access and awareness of antenatal care services in the country.

There is no documented significant association between age at marriage and perinatal depression in previous similar studies. However, in this study every one-year increase in age at marriage is independently associated with 1.2 times increase in perinatal depression. The reproductive age females may be depressed for being not married due to the social value given for marriage, the economic gain, and need of partner supports. Similarly, study participants who had health problems during their pregnancy had 5 times more likelihood of perinatal depression compared to mothers who do not experience health problems. This finding is consistent with previous studies conducted at South India and Japan [37, 39].

Study respondents who drink alcohol had 3.7 times lower odds of perinatal depression as compared to those who did not drink alcohol. On the contrary, husband smoking status is independently associated with about 4 times more likelihood of perinatal depression counterpointed to husbands not smoking. This finding is in line with the study conducted in South East Ethiopia [10].

Previous history of depression and family history of mental disorders were also independently associated factors with perinatal depression. Accordingly, respondents who had previous history of depression and family history of mental disorders were associated with 2.7 and 3.6 times more chance of having perinatal depression, respectively, as compared with their counter parts. Similarly, previous scholars had also reported consistent findings so far [10, 23, 25, 28–30, 34, 39]. This study was not free from limitations. First, it failed to assess factors such as food insecurity, current physical illness, and the hormonal/genetic factors which may importantly determine the perinatal depression. The cross-sectional nature of the design lacked the temporality between the factors and the outcome.

## 5. Conclusion

The study showed a high prevalence of perinatal depression among pregnant mothers and mothers who have less than a one-year-old child.

The independent factors associated with perinatal depression in this study were monthly income, number of pregnancy, age at marriage, health problems during pregnancy, alcohol use, husband smoking status, history of previous depression, and family history of psychiatric disorders.

## Figures and Tables

**Table 1:** Sociodemographic characteristics of participants Arba Minch Zuria District, Ethiopia, March, 2017.

Characteristics	Frequency (*n* = 704)	Percent
*Age group *		
15–24	192	27.27
25–34	384	54.55
35 and above	128	18.18
Age (mean ± SD)	28.02 ± 5.8	
Religion		
Protestant	510	72.44
Orthodox	186	26.42
Other	8	1.14
Ethnicity		
Gamo	465	66.05
Gofa	36	5.11
Wolayta	36	5.11
Amhara	105	14.91
Oromo	24	3.41
Other	38	5.40
*Marital status *(*n* = 702)		
Not married	24	3.42
Married	678	96.58
*Educational status *		
Unable to read and write	360	51.14
Primary education	234	33.24
Secondary education and above	110	15.63
*Occupation *		
Housewife	540	76.70
Farmer	98	13.92
Others	66	9.38
Husband occupation		
Farmer	482	69.05
Merchant	66	9.46
Daily laborer	92	13.18
Other	64	9.09
*Husband education *(*n* = 700)		
Unable to read and write	290	41.43
Primary Education	280	40.00
Secondary education and above	130	18.57
*Monthly income *(*n* = 694)		
Up to 1000	287	41.35
More than 1000	145	20.89
I do not know	262	37.75

**Table 2:** Substance abuse related characteristics of the respondents and their partners in Arba Minch Zuria District, Ethiopia, 2017.

Substance abuse characteristics	Frequency	Percent
Alcohol (*n* = 702)		
Yes	199	28.4
No	503	71.6
Husband alcohol (*n* = 698)		
Yes	313	44.8
No	385	55.2
Chat (*n* = 702)		
Yes	10	1.4
No	692	98.6
Husband chat (*n* = 698)		
Yes	14	2
No	684	98
Cigarette smoking (*n* = 704)		
Yes	14	2
No	690	98
Husband cigarette smoking (*n* = 700)		
Yes	60	8.6
No	640	91.4

**Table 3:** Analysis of Sociodemographic factors of participants associated with perinatal depression, Arba Minch Zuria District, Ethiopia, March, 2017.

Sociodemographic factors	Perinatal depression		COR^1^ (95% C.I)	*P* value	AOR^2^ (95% C.I)
	Yes	No			
*Age group *					
15–24	156	384	1		
25–34	20	78	.63 (0.37, 1.07)	0.086	
35 and above	12	54	.55 (0.28, 1.05)	0.070	
*Marital status *					
Not married	10	14	2 (.87, 4.6)	0.100	
Married	178	500	1		
*Educational status*					
Unable to read and write	118	242	2.86 (1.6, 5.1)	0.000^*∗*^	1.3 (0.5, 3.6)
Primary education	54	180	1.76 (0.96, 3.23)	0.07^*∗*^	
Secondary education and above	16	94	1		
*Occupation *					
Housewife	156	384	1.83 (0.95, 3.51)	0.07^*∗*^	1.5 (0.55, 3.9)
Farmer	20	78	1.15 (0.52, 2.56)	0.72	
Others^*∗∗*^	12	54	1		
*Husband occupation *					
Farmer	124	358	1		
Merchant	30	36	2.4 (1.4, 4)	0.001^*∗*^	
Daily laborer	26	66	1.14 (0.7, 1.8)	0.6	
Other^3^	8	56	0.4 (0.2, 0.9)	0.024^*∗*^	
*Husband education *					
Unable to read and write	84	206	1.8 (1.1, 3)	0.024^*∗*^	1.2 (0.5, 2.8)
Primary education	78	202	1.7 (1.02, 2.85)	0.04^*∗*^	
Secondary education and above	24	106	1		
*Monthly income *					
Up to 1000	64	223	2 (1.15, 3.6)	0.015^*∗*^	4.2 (1.9,9.3)^*∗∗*^
More than 1000	18	127	1		
I do not know	104	158	4.6 (2.7, 8)	0.000	5.9 (2.6,13.5)^*∗∗*^

^*∗*^Significant association in the bivariate analysis. ^*∗∗*^Significant association in the multivariable analysis. ^1^Crude Odds Ratio. ^2^Adjusted Odds Ratio. ^3^Government employee, jobless.

**Table 4:** Analysis of obstetric factors of participants associated with perinatal depression, Arba Minch Zuria District, Ethiopia, March, 2017.

Obstetric factors	Perinatal depression		COR (95% C.I)	*P* value	AOR (95% C.I)
	Yes	No			
*Number of pregnancy *					
Primigravida	12	107	0.16 (0.08, 0.31)	0.000^*∗*^	0.14 (0.03,0.65)^*∗∗*^
Multigravida	84	278	0.43 (0.3, 0.6)	0.000	0.5 (0.24,0.99)^*∗∗*^
Grand multigravida	90	129	1		
*Number of children *					
No child	2	61	0.05 (0.01, 0.22)	0.000^*∗*^	0.36 (0.05, 2.6)
1 child	26	110	0.37 (0.2, 0.66)	0.001^*∗*^	1.23 (0.37, 4.1)
2–4 children	122	280	0.68 (0.43, 0.97)	0.096^*∗*^	1.17 (0.5, 2.67)
More than four children	38	59	1		
Age at marriage			1.1 (1.05, 1.17)	0.000^*∗*^	1.2 (1.1,1.3)^*∗∗*^
Abortion					
Yes	54	71	2.54 (1.7, 3.8)	0.000^*∗*^	1.23 (0.66, 2.3)
No	132	441	1		
Health problems during pregnancy					
Yes	64	49	4.9 (3.2, 7.5)	0.000^*∗*^	5 (2.5,10.4)^*∗∗*^
No	124	465			
Planned pregnancy					
Yes	142	434	0.58 (0.39, 0.88)	0.01^*∗*^	1.12 (0.6, 2.14)
No	46	82			
ANC					
Regular	162	452	0.88 (0.54, 1.44)	0.62	
Sometimes/never	26	64			
Child death					
Yes	48	64	2.3 (1.5, 3.5)	0.000^*∗*^	0.86 (0.44, 1.67)
No	138	419	1		
Child hospitalization					
Yes	16	34	1.2 (0.65, 2.26)	0.54	
No	170	439	1		
Mode of delivery					
Vaginal	176	423	1.25 (0.6, 2.6)	0.55	
C/S Instrumental	10	30			
Child sex					
Male	102	237	1.08 (0.77, 1.53)	0.64	
Female	84	212			

^*∗*^Significant association in the bivariate analysis. ^*∗∗*^Significant association in the multivariable analysis.

**Table 5:** Analysis of substance abuse related factors of participants associated with perinatal depression, Arba Minch Zuria District, Ethiopia, March, 2017.

Substance abuse related factors	Perinatal depression		COR (95% C.I)	*P* value	AOR (95% C.I)
	Yes	No			
*Alcohol *					
Yes	40	159	0.6 (0.4, 0.9)	0.012^*∗*^	0.27 (0.14,0.52)^*∗∗*^
No	148	355	1		
Husband alcohol					
Yes	80	233	0.9 (0.64, 1.27)	0.56	
No	106	279			
Chat chewing					
Yes	2	8	0.68 (0.14, 3.23)	0.63	
No	186	506			
Husband chat					
Yes	8	6	3.8 (1.3, 11)	0.015^*∗*^	
No	178	506			
Smoking					
Yes	6	8	2.1 (0.72, 6.1)	0.18	
No	182	508			
Husband smoking					4.12 (1.6,10.6)^*∗∗*^
Yes	32	28	3.6 (2.1, 6.2)	0.000^*∗*^	
No	154	486			

^*∗*^Significant association in the bivariate analysis. ^*∗∗*^Significant association in the multivariable analysis.

**Table 6:** Analysis of previous history of mental disorders factors of participants associated with perinatal depression, Arba Minch Zuria District, Ethiopia, March, 2017.

Previous history	Perinatal depression		COR (95% C.I)	*P* value	AOR (95% C.I)
	Yes	No			
*Previous history of depression *					
Yes	76	83	3.54 (2.44, 5.15)	0.000^*∗*^	2.7 (1.54,4.84)^*∗∗*^
No	112	433			
*Family member mental disorder *					
Near relative	16	14	3.6 (1.73, 7.19)	0.001^*∗*^	3.6 (1.4,9.1)^*∗∗*^
Distant relative	16	10	5.1 (2.25, 11.4)	0.000	1.7 (0.57, 5.12)
No family member with mental disorder	154	488	1		

^*∗*^Significant association in the bivariate analysis. ^*∗∗*^Significant association in the multivariable analysis.

**Table 7:** Bivariate analysis of social support related factors of participants associated with perinatal depression, Arba Minch Zuria District, Ethiopia, March, 2017.

Social support factors	Perinatal depression		COR (95% C.I)	*P* value	AOR (95% C.I)
	Yes	No			
*Abuse*					
Yes	56	108	1.6 (1.1, 2.3)	0.014^*∗*^	1.6 (0.88, 2.9)
No	132	408			
Happy relationship					
* Yes*	160	444	0.99 (0.62, 1.62)	0.99	
* No*	26	72			
Husband feeling					
Very good	102	324	0.35 (0.21, 0.58)	0.000^*∗*^	0.96 (0.4, 2.3)
Good	48	152	0.35 (0.20, 0.61)	0.000^*∗*^	0.97 (0.4, 2.4)
Not good	36	40	1		
Difficulty of child caring					
Yes	56	54	3.62 (2.4, 5.5)	0.000^*∗*^	0.86 (0.46, 1.63)
No	130	454			
Husband support					
Yes	138	440	0.43 (0.28, 0.66)	0.000^*∗*^	0.64 (0.32, 1.27)
No	44	60			
Family presence during delivery					
Yes	162	429	0.47 (0.27, 0.83)	0.009^*∗*^	0.63 (0.28, 1.44)
No	24	30			
Happy in laws					
Yes	166	463	0.88 (0.51, 1.52)	0.64	
No	20	49			

^*∗*^Significant association.

## Abbreviations

HDSS: Health and Demographic Surveillance Site
EPDS: Edinburgh Postnatal Depression Scale
PND: Perinatal depression
SRQ: Self-reported questionnaire
WHO: World Health Organization.
